# Mutational spectrum of breast cancer by shallow whole-genome sequencing of cfDNA and tumor gene panel analysis

**DOI:** 10.1371/journal.pone.0308176

**Published:** 2024-09-12

**Authors:** Fernando Ambriz-Barrera, Ernesto Rojas-Jiménez, Clara Estela Díaz-Velásquez, Aldo Hugo De-La-Cruz-Montoya, Héctor Martínez-Gregorio, Miguel Ruiz-De-La-Cruz, Antonio Huertas, Ana Lorena Montealegre, Carlos Castro-Rojas, Gabriela Acosta, Felipe Vaca-Paniagua, Sandra Perdomo

**Affiliations:** 1 Laboratorio Nacional en Salud, Diagnóstico Molecular y Efecto Ambiental en Enfermedades Crónico-Degenerativas, Facultad de Estudios Superiores Iztacala, Tlalnepantla, México; 2 Unidad de Investigación en Biomedicina, Facultad de Estudios Superiores Iztacala, UNAM, Tlalnepantla, México; 3 Terry Fox National Tumor Bank, Instituto Nacional de Cancerología, Bogotá, Colombia; 4 Nutrition, Genetics and Metabolism Research Group, Faculty of Medicine, Universidad El Bosque, Bogotá, Colombia; 5 Genomic Epidemiology Branch, International Agency for Research on Cancer (IARC/WHO), Lyon, France; Nelson Mandela African Institute of Science and Technology, TANZANIA, UNITED REPUBLIC OF

## Abstract

Breast cancer (BC) has different molecular subgroups related to different risks and treatments. Tumor biopsies for BC detection are invasive and may not reflect tumor heterogeneity. Liquid biopsies have become relevant because they might overcome these limitations. We rationalize that liquid cfDNA biopsies through shallow whole genome sequencing (sWGS) could improve the detection of tumor alterations, complementing the genomic profiling. We evaluated the feasibility to detect somatic copy number alterations (SCNAs) in BC using shallow whole genome sequencing (sWGS) in cfDNA from archived samples from National Cancer Institute of Colombia patients. We sequenced tumor tissues from 38 BC patients with different molecular subtypes using a gene panel of 176 genes significantly mutated in cancer, and by liquid biopsies using sWGS on 20 paired samples to detect SCNAs and compare with the tumor samples. We identified an extensive intertumoral heterogeneity between the molecular subtypes of BC, with a mean tumor load of 602 mutations in the gene panel of tumor tissues. There was a 12.3% of concordance in deletions in the cfDNA-tumor pairs considering only the genes covered by the panel encompassing seven genes: *BRCA1*, *CDK12*, *NF1*, *MAP2K4*, *NCOR1*, *TP53*, and *KEAP1* in three patients. This study shows the feasibility to complement the genomic analysis of tumor tissue biopsies to detect SCNA in BC using sWGS in cfDNA, providing a wider identification of potential therapeutic targets.

## Introduction

Breast cancer (BC) is a heterogeneous disease comprising several molecular subtypes, with different risk profiles and treatments [1]. Worldwide the incidence and mortality rates (age-standardized) of BC in women are 47.8 and 13.6, respectively [2]. The number of newly diagnosed BC is projected to grow by over 40% in 2040. A large relative increase will be particularly seen in transitioning countries including Latin America [3]. A large proportion BC cases in Latin American countries are diagnosed at advanced stages, and only 10–20% at early stages, contrasting with the United States, where 60% are diagnosed early, resulting in an incidence-mortality ratio of 0.59 for Latin America compared to 0.43 for the European Union, and 0.35 for the United States [4, 5]. On the other hand, tumor biopsies are frequently used for pathological characterization and selection of treatment [6]. The urgent need to increase early BC detection requires the implementation of improved diagnostic techniques. However, the use of tumor tissues has several limitations due: i) early stages tumors biopsies sampling are limited; ii) and might not reflect the intratumor heterogeneity of tumor; iii) they are also highly invasive techniques, iv) high costs. The use of minimally invasive techniques such as liquid biopsies to evaluation of cell-free DNA (cfDNA), are an alternative to tumor tissues, because they are less invasive and can better capture the heterogeneity seen in the tumor [7, 8]. cfDNA are 150–200 bp fragments of DNA from tumor and normal tissues released into the bloodstream or other biological fluids through necrotic, apoptotic and cellular secretion processes with an average duration in the bloodstream of 16–250 min [9–11]. Several studies in BC have demonstrated the utility of cfDNA evaluation for early detection [12], monitoring of minimal residual disease [13, 14], identifying clonal evolution [15] and tumor heterogeneity [16], and predicting tumor progression [17], immune response [18], mechanisms of resistance to treatment [19] and metastatic relapse [9, 20, 21]. The ability to identify single nucleotide variants (SNVs) and somatic copy number alterations (SCNAs) in cfDNA might overcome the challenges faced by detection of these alterations using whole genome sequencing in tumor samples due to cost and storage of the data generated. In addition, genomic analyses in cfDNA can improve tumor molecular classification at diagnosis and support the selection of tailored treatments [22, 23]. Several groups have evaluated the use of shallow whole-genome sequencing (sWGS) to identify SCNAs in several tumors including BC as a less expensive alternative [24, 25]. sWGS is a high-throughput technology that provides a cost-effective and accurate method to achieve genome-wide genetic variation with very low coverage (most frequently between 0.4x and 1x) [26]. For this reason, we explored, as a proof of principle, the use of sWGS and targeted sequencing as a testing strategy to identify the genomic profile of plasma and tumor samples from BC patients. We sequenced 38 tumor tissues of BC patients using a panel of 176 cancer driver genes to identify SNVs and SCNAs, and cfDNA from 20 paired plasma samples to identify SCNAs using sWGS. This study shows the feasibility to complement the genomic analysis of tumor tissue biopsies to detect SCNA in BC using sWGS in cfDNA, providing a wider identification of potential therapeutic targets.

## Materials and methods

### Patient selection

**Fig 1:**
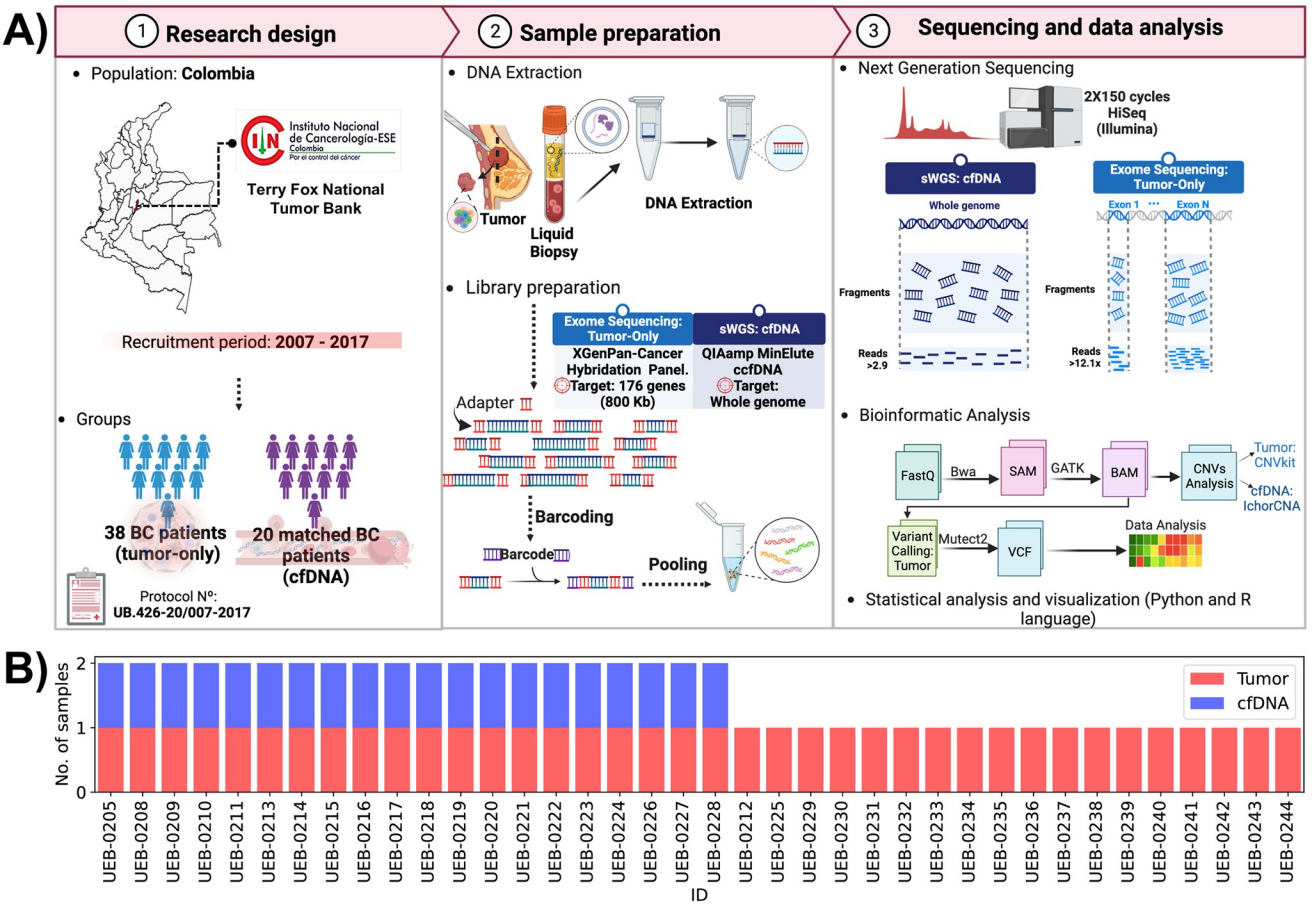
(A) Experimental design. The workflow from sample recruitment to sequencing data analysis is shown. Panel 1. Sample selection. Thirty-eight BC patients were selected from the Terry Fox National Tumor Bank (National Cancer Institute in Colombia) diagnosed in a period of 10 years (2007–2017). Panel 2. Sample preparation and barcoding. Genomic DNA was extracted by affinity column followed by library preparation using exome enrichment (176 genes for tumor samples) and shallow whole genome sequencing for cfDNA. The samples were sequenced on an Illumina HiSeq in a 2X150 cycle format. Panel 3. Sequencing and data analysis. Identification of SCNAs in both tumor and cfDNA, and SNV in tumor tissues. BC: Breast Cancer; cfDNA: circulating free DNA; sWGS: Shallow Whole Genome Sequencing; SCNA: Somatic Copy Number alteration; SNV: Single Nucleotide Variant. (B) Samples used for this study. cfDNA samples obtained from plasma are depicted in blue, and tumor samples are shown in red. Created with Biorender.

We included 38 patients diagnosed with BC and treated at the National Cancer Institute between 2007 and 2017, who consented to donate their samples to the Terry Fox National Tumor Bank in Bogota, Colombia (Fig 1). Clinical classification of BC was defined by immunohistochemistry (IHC) based on the established criteria of positivity for the ER, PR, and HER2 receptors. HER2 positivity was evaluated initially by IHC if circumferential membrane staining was complete, intense and in > 10% of tumor cells. HER2 ISH was complementary used if the IHC result was +2 equivocal according to the College of American Pathologists (CAP) guidelines [27]. Tumor and plasma samples from selected patients were classified as primary (treatment naive) and treated according to the point in time in which they were collected. In tumor samples, 22 were primary treatment-naive, 15 were collected after treatments and one did not have information on date of collection, whereas in cfDNA samples 13 were collected before any documented treatment, six after treatment and 1 did not have information on date of collection (S1 Table in S1 File). The study was conducted according to the guidelines of the Declaration of Helsinki, and was approved by the Ethics Committee of the National Cancer Institute of Colombia (National tumor bank Terry Fox acta 004–2017) and El Bosque University (protocol UB.426–2016/007-2017). The Statement of Informed Consent was obtained from the patient/study participants at the Terry Fox National Tumor Bank-National Cancer Institute of Colombia Data Availability. Medical records were accesed on December 2, 2022. No minors were included in this work.

### DNA extraction of tumor tissue and cfDNA

Five mL of whole blood was collected in EDTA tubes. Two mL of blood were used for plasma obtention by centrifuge fractioning with Ficoll gradient. Plasma was frozen at -70ºC until use. Fresh frozen tumor tissues were obtained from the Terry Fox National Tumor Bank in Colombia. All the tissues had a tumor cellularity of 70% and were excised. DNA of tumor tissues were extracted with DNeasy Blood & Tissue Kit (Qiagen, Hilden, Germany) and the plasma DNA with the QIAamp ccfDNA kit (Qiagen, Hilden, Germany), following the manufacturer’s instructions. DNA concentration was quantified by Qubit (Invitrogen, Carlsbad, USA). The integrity and purity were verified by agarose gel electrophoresis and spectrophotometry, respectively.

### Library preparation and sequencing

The library preparation of tumor tissues was done with the xGenTM Pan-Cancer Hybridation Panel (IDT, Coralville, Iowa, USA), which covers 176 genes significantly mutated in cancer (800 Kb of protein-coding bases) (S2 Table in S1 File). The cfDNA libraries for the sWGS analysis were done with the QIAamp MinElute ccfDNA Kit (Qiagen Hilden, Germany), following the manufacturer’s instructions. All the libraries were prepared at the National Health Laboratory of the Faculty of Higher Studies, Iztacala, UNAM, Mexico. Pair-end sequencing was performed on an Illumina HiSeq 2500 for 2X150 cycles at Novogen, Sacramento, California.

### Bioinformatic analysis of SNV in tumor tissues

Bioinformatic preprocessing were performed using previously described methods (S3 Fig in S1 File) [28, 29]. Briefly, BWA [30] and GATK [31] were used for alignment and data processing, respectively. We used Mutect2 in tumor-only mode for variant calling and Annovar [32] for annotations. Variants were filtered as follows: i) variants with quality above 30, ii) with an allelic frequency of less than 0.001 in 1000 genomes, ExAC, and ESP6500, iii) with an allelic fraction of ≥ 0.03, and iv) with at least two reads on both DNA strands were kept. Later, driver genes were selected using Intogen v.2020 list [33]. Deleterious variants were defined if two out of three algorithms—SIFT, PolyPhen2, and MutationTaster—predicted the variant as deleterious. In addition, driver genes were classified in Tier I-IV following the recommendations of American Society of Clinical Oncology and College of American Pathologists (ASCO/CAP) [34]. The levels of clinical evidence were obtained from oncoKB [35] and Cancer Genome Interpreter [36]. All driver variants were manually curated by inspection of the BAM files using the IGV software [37].

### Detection of SCNA in tumor tissues and cfDNA

CNVkit [38] was used to detect SCNAs in tumor samples with default parameters using the tumor-only mode. SCNAs were filtered considering the biological function of the gene affected as follows: i) SCNAs with CN = 2 were excluded, ii) Oncogenes with CN<2 were excluded and CN>2 were kept, iii) tumor suppressor genes with CN>2 were excluded and CN<2 were kept. Regions with a depth <10X were excluded. Selection of driver genes based on SCNAs followed Intogen v.2020 list [33] and pathogenicity evaluation with tier classification using oncoKB [35] and Cancer Genome Interpreter [36] following the recommendations of American Society of Clinical Oncology and College of American Pathologists (ASCO/CAP). To classify the potential clinical effects of these variants, the SCNA loss events were considered as loss of function and the SCNA gain events as gain of function. We compared three different methods for SCNA calling in cfDNA (ichorCNA, QDNAseq and Control-FREEC) at different window sizes (15Kb, 50kb, 500kb, 1Mb) and parameters (segmentation with log2 or sqrt transformation for QDNA, GEM mapping for Control-FREEC, self-transition probability of 0.9999999 and transition pseudo-counts of 10000 for ichorCNA) (S4 Fig in S1 File). The results of this analysis showed that among the three programs, ichorCNA had the highest sensitivity and specificity, the lowest rate of false positives in previously described simulated tests [39], and presented more similarity between cfDNA and tumor DNA in SCNA S5 Fig in S1 File. We used ichorCNA to detect SCNA in cfDNA with a bin size of 500kb [24] and default parameters. SCNAs were annotated in AnnotSV [40] and filtered by the criteria previously mentioned. The regions of log2 copy < |0.3| were excluded to improve variant purity and reliability. Subsequently, we evaluated the status of homologous recombination deficiency using shallowHRD [41] based on the recommended parameters, with a threshold of >15 large-scale genomic alteration (LGA) events to define HRD positivity.

### Statistical analysis

The evaluation of significant differences between cfDNA concentration and clinical characteristics were performed by Kruskal-Wallis or ANOVA in R v.4.1.2 [42] and python v3.10.6 [43] using the packages stats and scikit-learn [44], respectively. Association of cfDNA concentration with age was evaluated with numpy [45] to a polynomial regression and plotted with seaborn [46]. Pearson correlation was used to evaluate inter-sample swapping with the library base of R, considering r>0.8 as high correlation.

## Results

### Clinical characteristics

We studied 38 patients diagnosed with BC treated at the National Cancer Institute in Colombia that gave consent to donate biological samples to the Terry Fox National Tumor Bank between 2007 and 2017. The patients showed different molecular subtypes, clinical scenarios, and treatment responses (Table 1). The mean age of patients was 57.68 years (± 13.09), 35 were females (92.1%) and 3 males (7.9%). The BC molecular subtypes were distributed as follows: 42.1% luminal A, 26.3% luminal B like HER2+, 10.5% triple negative, 5.3% luminal B, 5.3% HER2+, and 10.5% unknown. Based on histological subtype, there were 28 patients (73.7%) with invasive ductal carcinoma (IDC), 4 (10.5%) with phyllodes tumor, 2 (5.3%) with lobular carcinoma, 2 (5.3%) with metaplastic carcinoma, and 2 (5.3%) with mucinous carcinoma. Clinical staging was characterized by local disease with 4 patients (10.5%) diagnosed in stage I, 16 (42.2%) in stage II, and 14 (35.9%) in stage III. Staging information was missing in 4 (10.5%) cases.

**Table 1:** Clinical and epidemiological characteristics in our cohort of study.

Age	Mean±SD	n (%)	Treatment	n	%
<50 years	43.93.1±5.48	13 (34.21)	Surgery	8	21.1
>50 years	64.84±9.66	25 (65.78)	Radiotherapy	4	10.5
Overall	57.68± 13.09	38 (100)	Chemotherapy	4	10.5
			Rad-Chem	22	57.9
Sample:tumor	n = 38	%	Sample:cfDNA	n = 20	%
P. treatment-naive tumor	22	57.9	P. treatment-naive tumor	13	65
Treated tumor	15	39.4	Treated tumor	6	30
Unknown	1	2.6	Unknown	1	5
BMI	Mean±SD	n(%)	Histological subtype	n	%
<18.5 underweight	N/A	1 (2.63)	IDC	28	73.7
18 < 25 normal weight	22.35±3.95	8 (21.1)	Lobular	2	5.3
25 < 30 overweight	27.21±4.3	11 (28.95)	Metaplastic	2	5.3
30 < 40 obese	32.99±4.81	3 (7.89)	Mucinous	2	5.3
Unknown	N/A	15 (39.47)	Phyllodes	4	10.5
Sex	n	%	Family history of cancer	n	%
Female	35	92.1	Yes	14	36.8
Male	3	7.9	No	9	23.7
			Unknown	15	39.5
Molecular subtype	n	%	Stage	n	%
Luminal A	16	42.1	IA	4	10.5
Luminal B	2	5.3	IIA	8	21.1
Luminal B Like HER2+	10	26.3	IIB	8	21.1
HER2+	2	5.3	IIIA	2	5.3
Triple Negative	4	10.5	IIIB	10	26.3
Unknown	4	10.5	IIIC	2	5.3
			Unknown	4	10.5
Ki67 level	Mean±SD	n (%)	TNM classification	n	%
			T0	1	2.6
1–5%	2.5±0.24	2 (5.3)	T1	5	13.2
6–15%	10.5±0.22	5 (13.2)	T2	15	39.5
>15%	31.08±0.22	23 (60.5)	T3	3	7.9
Unknown	N/A	8 (21.1)	T4	11	28.9
			Unknown	3	7.9
PR status	n	%	N	n	%
			N0	16	42.1
Positive	24	63.2	N1	12	31.6
Negative	11	28.9	N2	5	13.2
Unknown	3	7.9	N3	2	5.3
			Unknown	3	7.9
HER2 status	n	%	M	n	%
Positive	12	31.6	M0	35	92.1
Negative	22	57.9	M1	0	0
Unknown	4	10.5	Unknown	3	7.9
ER status	n	%	Vital status	n	%
Positive	27	71.1	Alive	32	84.2
Negative	8	21.1	Dead	1	2.6
Unknown	3	7.9	Unknown	5	13.2

### Breast cancer showed intertumoral heterogeneity at the level of SNV

**Fig 2:**
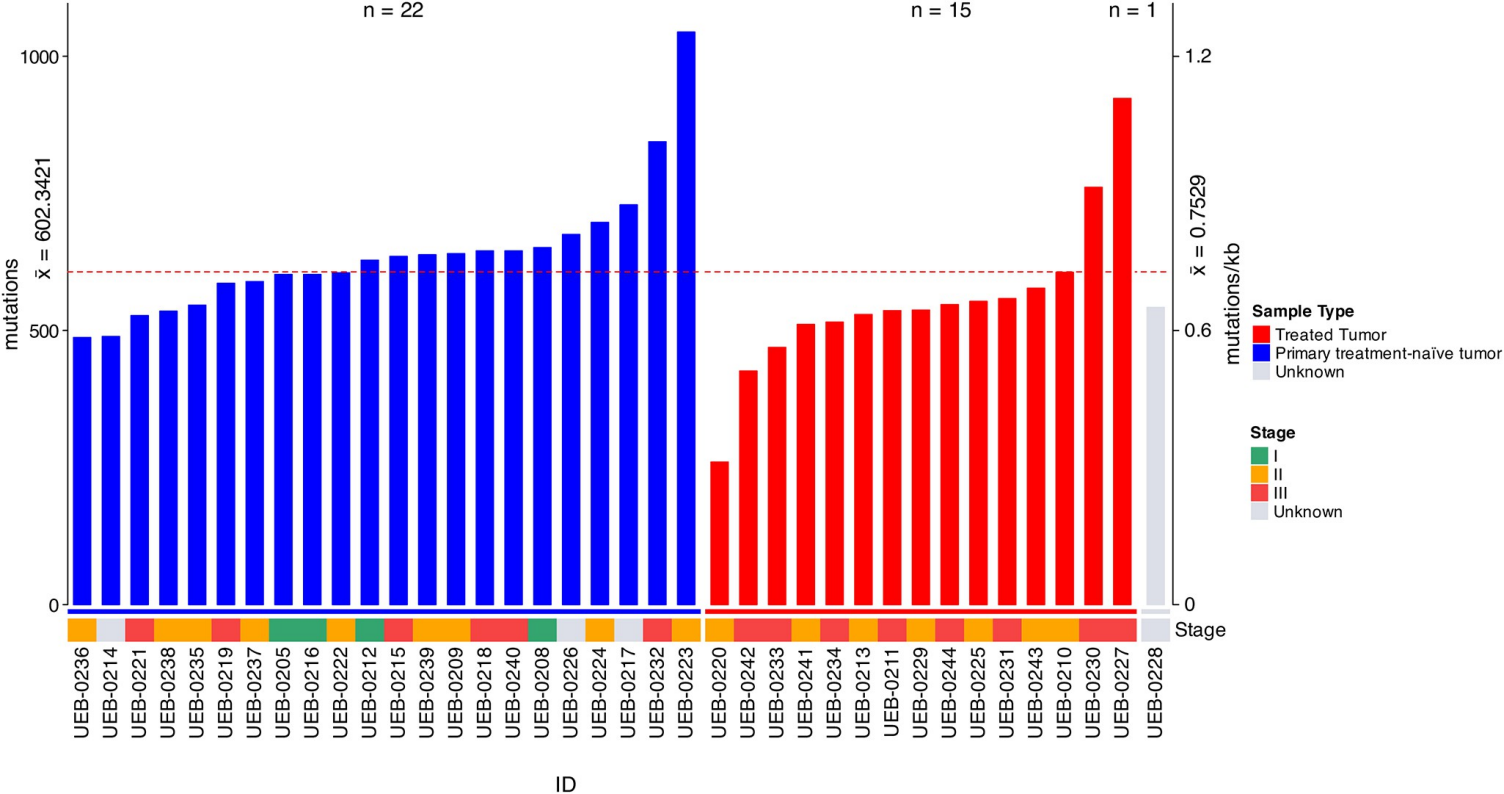
Tumor mutation rate in 38 breast cancer tumor tissues. Number of mutations segmented by sample type in ascending order. The clinical stages of these tumors are shown in the lower track. The horizontal, dotted line represents the mean mutational load.

Thirty-eight BC tumor tissues were sequenced with a panel of 176 cancer driver genes to assess the mutational landscape of BC. To control for inter-patient swapping and batch effects, we first evaluated inter-sample similarity by a Pearson analysis with the dichotomic SCNA events. We used the SCNA data from the cfDNA and tumor samples. The maximum correlation was r = 0.6, indicating a lack of correlation S6 Fig in S1 File. The mean sequencing depth was 186X (±47.97). We found an extensive genetic interpatient heterogeneity across the different molecular subtypes of BC. In tumor samples a total of 17,415 SNVs, including 8,367 synonymous, 8,046 non-synonymous mutations and 1,002 indels were identified. We detected a mean mutation of 602 with a tumor mutational burden of 0.7529 mutations/kb (range 0.32–1.3), (Fig 2).

**Fig 3:**
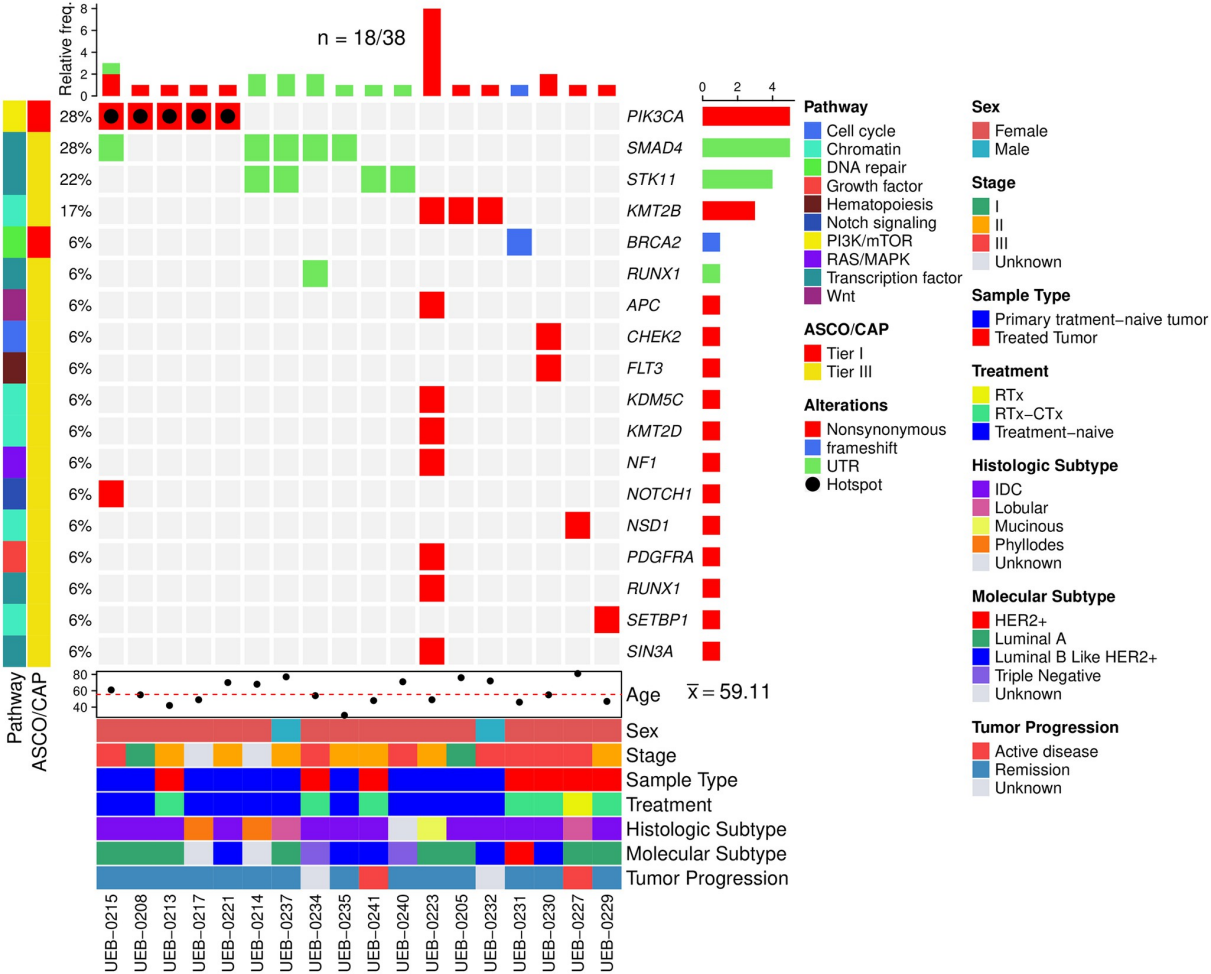
Pathogenic variants and VUS identified in 38 breast cancer patients. The total number of driver genes with variants in each sample are shown in the top panel. Signaling pathways and classification of variants following the recommendations of American Society of Clinical Oncology and College of American Pathologists (ASCO/CAP) are shown in the left panel. The clinical characteristics are shown in the bottom panel. UTR: untranslated region; IDC: intraductal carcinoma; CTx: chemotherapy; RTx: radiotherapy; Sx: surgery.

Eighteen out of 38 patients (47.4%) had at least one pathogenic/likely pathogenic variant or a variant of unknown significance (VUS) in 18 driver genes (Fig 3). Two pathogenic variants were detected in *PIK3CA* and *BRCA2*, while the rest of patients had at least one VUS in the remaining 16 driver genes. *PIK3CA* hotspot mutation p.H1047R was observed in five patients (13%) diagnosed with different molecular subtypes: three luminal A patients, one luminal B like HER2+, and one patient missing molecular classification. The second variant, *BRCA2* p.Q2009Afs*8 was found in one patient with a HER2+ tumor (3%). The gene ontology analysis revealed enrichment in 10 important signaling pathways: transcription factors (28%), chromatin remodeling (28%), cell cycle, DNA repair, growth factor, hematopoiesis, Notch signaling, PI3K/mTOR, RAS/MAPK, and Wnt, each with 6%.

### Changes in cfDNA concentration with clinical variables

**Fig 4:**
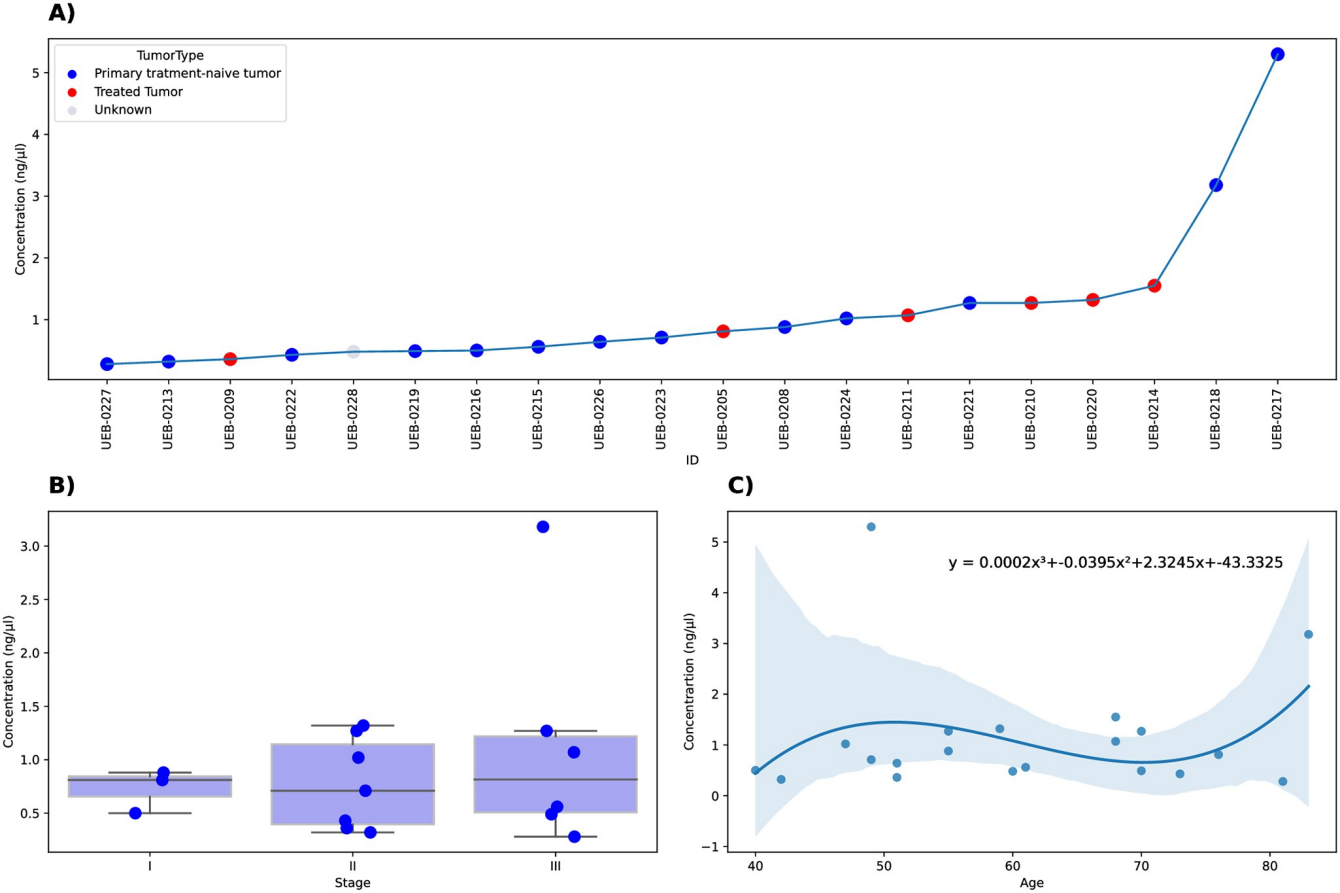
cfDNA concentration between the groups with different stages and treatments. (A) cfDNA concentration per patient in ascending order, (B) cfDNA concentration by different stages of the disease (ANOVA test, p = 0.60), and (C) distribution of cfDNA concentration and age.

Plasma samples were available for 20/38 BC patients. We measured and compared cfDNA concentration levels between patients with different clinical stages or treatments to analyze the reported association of cfDNA levels with those clinical variables. The mean concentration of cfDNA was 1.1 ng/uL (range: 0.28–5.30). Only two patients, both with primary treatment-naive tumors (UEB-0218 and UEB-0217), had higher cfDNA concentration (3.2 ng/uL and 5.3 ng/uL, respectively), while the rest (90% of samples) less than 2 ng/uL (Fig 4A). We didn’t find statistical differences between mean cfDNA concentration and cancer stage (mean 0.73 ng/uL in stage I, 0.77 ng/uL in stage II, and 1.14 ng/uL in stage III; ANOVA, p = 0.60; Fig 4B). There was no correlation between cfDNA concentration and age (Fig 4C). The analysis to detect homologous recombination deficiency was negative for all samples. However, UEB-0218 had borderline (≥15 LGAs, see Materials and methods) results for this classification, presenting 15 LGAs (S1 Fig in S1 File).

### cfDNA revealed a higher number of SCNAs in comparison to tumor tissue biopsies

**Fig 5:**
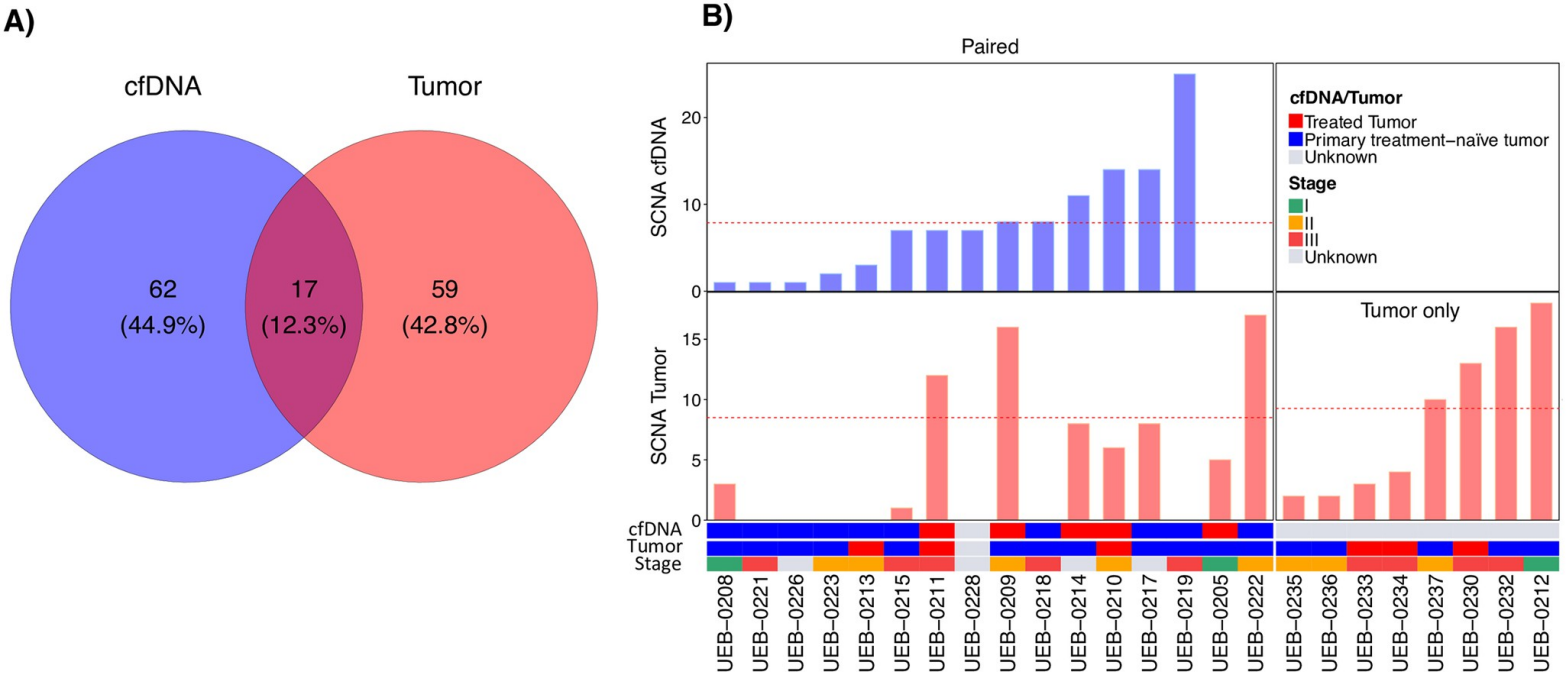
Total somatic copy number alterations events identified in tumor tissues and cfDNA. (A) Venn diagram depicting the differences and similarity of the SCNAs detected in cfDNA (blue) and tumor tissues (orange). For this analysis only the paired samples were considered (cfDNA-tumor tissues). (B) Number of SCNAs identified in cfDNA and tumor tissues by patient.

The mean depth of whole genome cfDNA sequencing was 2.9X (±0.17, range: 2.65–3.28X) and the mean tumor fraction was 0.014 (0–0.02) (S2 Fig in S1 File). We evaluated the SCNA events in 18 tumors alone, and 20 tumor:cfDNA paired samples. Subsequently, in the paired samples we compared the concordance between the SCNA events detected in the tumors and those from the cfDNA considering the 176 gene panel used for the tumor sequencing (see Material and methods). Overall, 138 SCNAs were detected. Sixty-two (45%) SCNAs were exclusively found in cfDNA, 59 (43%) in the tumor tissues, and 17 (12%) SCNAs were common (Fig 5A). Twenty-one (55.3%) did not show SCNAs in tumor tissues and 6 (30%) samples did not show SCNAs in cfDNA (Fig 5B).

**Fig 6:**
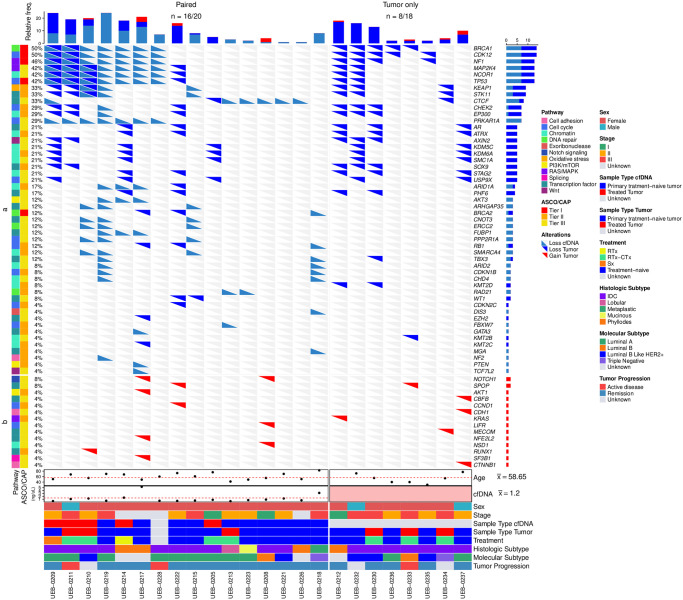
Tumor and cfDNA SCNA events in driver genes and clinical characteristics in the cohort. Samples are presented in two groups, the cfDNA:tumor pairs on the left, and tumor only on the right. The alterations in the cfDNA and tumor samples are illustrated as left and right flags, respectively. The number of affected driver genes in each sample is shown in the top panel. The ASCO/CAP classification, mutation signaling pathways, and relative frequency are shown on the left side. The absolute frequency of SCNA events per gene and the legends are shown on the right. Clinical characteristics are shown in the bottom panel.

We detected SCNAs in 64 genes. The genes with higher relative frequency of SCNAs in the cfDNA and the tumor samples were: *BRCA1*(50%), *CDK12* (50%), *NF1* (46%), *MAP2K4*(42%), *NCOR1* (42%), *TP53* (42%), *KEAP1* (33%), *STK11* (33%), *CTCF* (33%), *CHEK2* (29%), *EP300* (29%), and *PRKAR1A* (29%) (Fig 6). The genes *BRCA1*, *CDK12*, *NF1*, *MAP2K4*, *NCOR1*, *TP53* and *PRKAR1A*, from chromosome 17 had the highest number of SCNA events. In the 20 paired samples, two patients (UEB-0209 and UEB-0211) had tumor/cfDNA shared deletions in *BRCA1* and *CDK12*; three (UEB-0209, UEB-0211, and UEB-0210) in *NF1*, *MAP2K4*, *NCOR1*, and *TP53*; and one (UEB-0210) in *KEAP1*. Seven patients (UEB-0219, UEB-0228, UEB-0213, UEB-0223, UEB-0221, UEB-0226, and UEB-218) had SCNA events that were not detected in tumor tissue; and two patients (UEB-0205 and UEB-0222) presented SCNAs that were not detected in cfDNA. Only tumor samples exhibited amplifications. In the cfDNA samples of the HER2 subtypes (2 HER2+, and 10 Luminal B Like HER2+) we did not detect the amplification of the *ERBB2* locus. Based on ASCO/CAP classification, 35 (54%), 24 (35%) and 5 (7%) SCNA were classified as tier III, II and I, respectively. We identified five copy number deletions (affecting *BRCA1*, *BRCA2*, *CDK12*, *NF1*, and *TP53* genes) classified in tier I in both cfDNA and tumor tissues. The gene ontology analysis applied to the SCNA events revealed enrichment in 12 important signaling pathways: transcription factors (28%), chromatin (23%), cell cycle (18%), PI3K/mTOR (6%), DNA repair (6%), cell adhesion (5%), RAS/MAPK (5%), Wnt (3%), exoribonuclease (1.5%), oxidative stress (1.5%), Notch signaling (1.5%), and splicing (1.5%).

**Fig 7:**
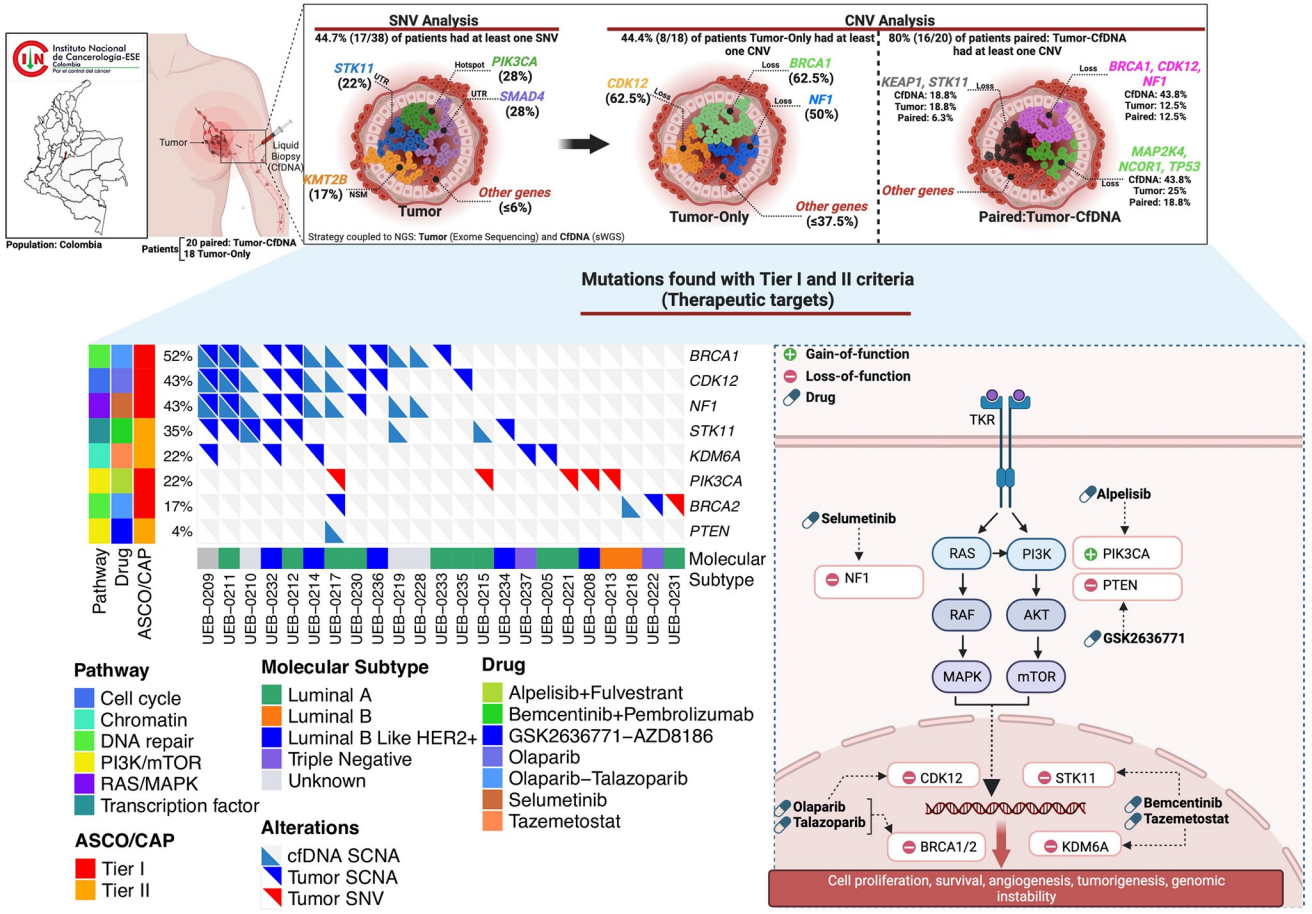
Alterations found according to ASCO/CAP classification in BC. The figure shows the different types of alterations (top), the existing therapeutic targets (left) and the principal pathways affected (right). Created with Biorender.

After integration of SNVs and SCNAs we identified 61 somatic alterations (24 in cfDNA and 37 in tumor tissue) in 8 genes according to ASCO/CAP classification to therapeutic targets (Fig 7). Cell cycle regulation and PIK3/mTOR signaling were the principal molecular pathways that could be therapeutically targeted in our study.

## Discussion

BC is a multifactorial complex disease that imposes a severe social, and economic burden with a projected increase in low and middle-income countries. For decades, the recommended scheme of early detection of BC has relied on mammography [47, 48]. However, the use of minimally invasive techniques, such as liquid biopsies, have recently become potentially relevant as it might overcome sampling limitations of traditional biopsies with similar benefits in terms of tumor molecular profiling [7, 8]. The feasibility of using this type of analyses in any clinical setting, however, should be carefully evaluated as differences might arise depending on variations in clinical or preclinical conditions. Therefore, we rationalized that liquid cfDNA biopsies through sWGS could improve the detection of tumor alterations, complementing the genomic profiling. The concentrations of cfDNA can be modified by different physiological conditions or clinical scenarios [49]. cfDNA has been used to detect and characterize circulating tumor DNA (ctDNA), allowing the integration of liquid biopsy into clinical practice for molecular profiling, serving as a promising biomarker for prognosis, monitoring the response to the disease, detecting minimal residual disease and early diagnosis [50]. We measured cfDNA concentration in 20 patients to evaluate the changes of cfDNA levels between the groups with distinct stages. Overall, we did not observe significant differences between stages. The lack of correlation between cfDNA concentration and stage or age might be due to the reduced number of evaluated patients. Moreover, the fluctuation of cfDNA concentration in the polynomial regression indicates interindividual differences influenced by treatment, stage, age, and molecular tumor type. An increase in the sample size could improve these results and clarify the different factors involved in the release of cfDNA. BC is characterized by a wide intertumoral and intratumoral heterogeneity [51, 52]. We evaluated the genomic profile of breast neoplasms by comparing SCNA from tumor tissues with cfDNA from plasma in paired samples and the SNV in the tumor samples. Intratumoral heterogeneity at the SNV level was similar to other previously reported studies in Latin America [28, 29]. To exclude the common genetic variation with neutral effects, we eliminated single nucleotide polymorphisms with the ExAC database. This database includes genetic information of 5789 exomes from Latin persons without oncological diseases. We found a mean somatic mutational load of 602 in 38 tumor tissues. This somatic mutational load reflects the limited region covered by the gene panel used (800 kb). We identified 47% patients with at least one pathogenic or likely pathogenic variant or VUS in 18 driver genes. Only two pathogenic variants were detected in *PIK3CA* (p.H1047R) and *BRCA2* (p.Q2009Afs*8) genes. Other studies have shown that luminal A has a prevalence of 49% in *PIK3CA* mutations [55], while *BRCA1* and *BRCA2* mutations are characteristic of triple negative breast cancer. The prevalence of *PIK3CA* variants in this study was 7.9%, however, we did not find SNV in *TP53*, which could be related to the small sample size. Gene ontology analysis revealed enrichment in several genes such as transcription factors, chromatin, cell cycle, DNA repair, and other, which are consistent with other studies from Latin America populations [53, 54]. Overall, SCNA events were more frequent (63%) in our patients in comparison to SNVs (47%). The most prevalent deletions occurred in key driver genes such as *BRCA1* (50%), *CDK12* (50%), *NF1* (46%), *MAP2K4* (42%), *NCOR1* (42%), *TP53* (42%), *KEAP1* (33%), *STK11* (33%), *CTCF* (33%), *CHEK2* (29%), *EP300* (29%), and *PRKAR1A* (29%). The loss of *TP53* and *BRCA1* results in chromosomal instability [55, 56], which is consistent with the higher contribution of SCNA rather than SNV to the genetic alterations of these tumors [52]. Similar findings have been shown in previous studies [57], and in Leukemia and prostate cancer, respectively [58, 59]. Additionally, SCNAs at gene loci contribute to most of the variations in gene expression of breast tumors and studies have suggested their potential implication in prognosis and overall survival [52]. In concordance with previous reports, the majority of SCNA events were found on chromosome 17, which has been widely reported to present chromosomal arm abnormalities in breast cancer [60]. We decided to focus on evaluating the concordance of SCNAs detection between tissue and liquid biopsies (plasma), using 20 cfDNA-tumor tissue pairs. We found a concordance of 12.3% in 7 genes: *BRCA1*, *CDK12*, *NF1*, *MAP2K4*, *NCOR1*, *TP53*, and *KEAP1*. In addition, there was no evidence of *ERBB2* amplification in the cfDNA from HER2+ tumors. This low level of concordance might be influenced by a low concentration of cfDNA and ctDNA fraction, the short half time of cfDNA [9], reduced tumor cellularity, and incomplete representation of the whole subclonal architecture of the tumor load, and SCNA at subclonal level (S1 Fig in S1 File). However, for those SCNAs identified, five clinically relevant alterations in *BRCA1*, *BRCA2*, *CDK12*, *NF1* and *TP53* genes were classified 24 (37.5%) in tier II and 35 (54.68%) in tier III according to ASCO/CAP criteria. Alterations in *BRCA1*, *BRCA2*, and *CDK12* are therapeutic targets to olaparib and talazoparib. Recent clinical trials have shown promising results for the use of these two PARP inhibitors in the treatment of BC. The OLYMPIA trial (NCT02032823) found that adjuvant olaparib for patients with *BRCA1*- or *BRCA2*-Mutated BC was associated with significantly longer survival free of invasive or distant disease than was placebo [61]. Olaparib is currently approved in Colombia for the treatment of BC patients. The EMBRACA trial (NCT01945775) found that single-agent talazoparib provided a significant benefit over standard chemotherapy with respect to progression-free survival among patients with advanced BC and a germline *BRCA1/2* mutation [40] and could be used as a potential BC treatment in those patients. Gene amplifications were only detected in tumor tissue. The lack of amplifications in cfDNA samples may be influenced by the reduced tumor fraction, even in cfDNA samples treatment naive. Moreover, cfDNA samples did not show homologous recombination deficiency due to low count of LGA events. To better capture the cfDNA tumor fraction and characterize more precisely the genomic alterations by sWGS, our design was considered to have deeper coverage of the target region of approximately 3X, as compared to previous studies which used 0.1X. Unexpectedly, our sequencing data revealed a lower tumor fraction (0.014) as compared to prior reports (0.03) [24], which may have reduced the sensitivity to detect arm-level SCNA. To reduce false positive rate, we calibrated the parameters using previously reported simulated data [39], improving sensitivity and specificity to capture the mutagenic profile of the tumor (S3–S5 Figs in S1 File). The tumor fraction has shown significant oscillations, which may account for the decreased level of this parameter despite a greater depth of coverage [24, 62]. In this work, we used the commercial design of the 176 gene panel for the tumor analysis based on their relevance and role in oncology from the TCGA project, however, does not include the *ERBB2* gene, therefore this amplification was not analyzed in the tumors. Our study demonstrated the capacity to perform an integrated genomic assessment of tumor and cfDNA pairs, confirming the feasibility of these analyzes in the context of BC. Although our ability to further evaluate clinical and genomic associations could be enhanced with a larger cohort. Our results reflect the limitation of real-world genomic data in the clinic, indicating that caution should be taken when implementing advanced genomic techniques in the clinic, as the ability to detect biomarkers may be compromised. However, we show that liquid biopsy can complement genetic analyzes performed on the tumor and improve the detection of SCNA that affect key tumor supressor genes, wich may reflect the intratumoral heterogeneity of the whole tumor load, providing additional potentially selectable markers thay may enhance the efficacy of treatments in the future.

## Conclusion

In conclusion, this work highlights the genomic complexity of BC and the feasibility to use cfDNA to complement the genomic analysis of tumor tissue biopsies for the detection of SCNA in key tumor supressor genes, providing a wider identification of potential actionable alterations that may guide to more effective therapeutic approaches.

## Supporting information

S1 File (PDF)
