# Supplementary material for: Mutational spectrum of breast cancer by shallow whole-genome sequencing of cfDNA and tumor gene panel analysis
Source: PLoS One. 2024 Sep 12;19(9):e0308176. doi: 10.1371/journal.pone.0308176 (PMC11392417; doi:10.1371/journal.pone.0308176)
Supplement: S1 File — (PDF) [file pone.0308176.s001.pdf]

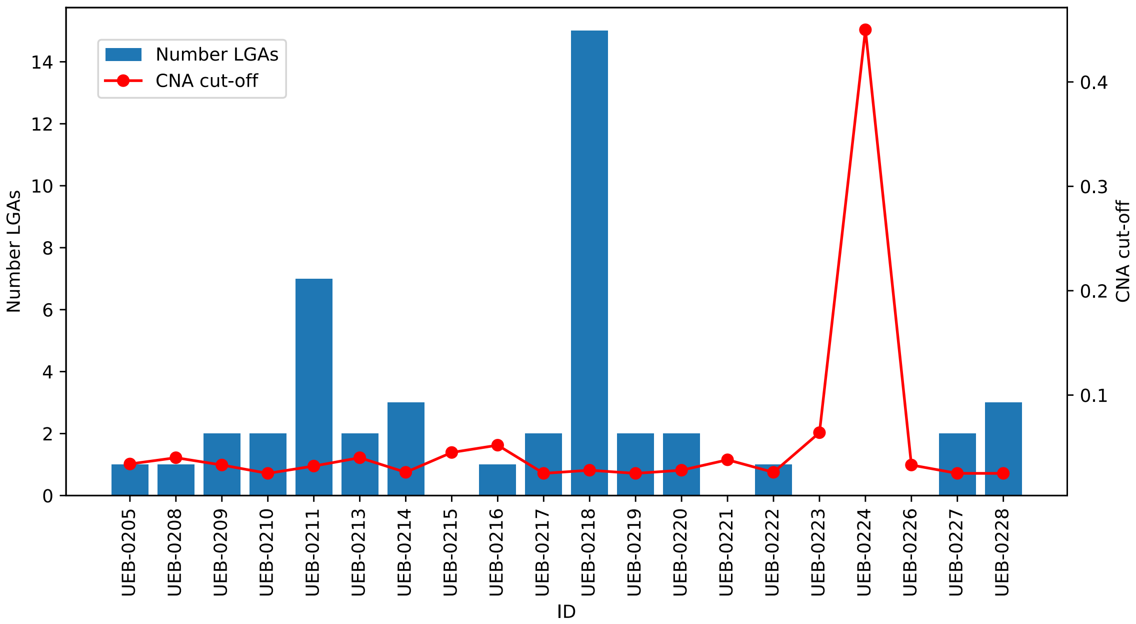

Figure S1. Analysis for homologous recombination detection in shallow WGS in breast cancer patients. The figure shows the number of large-scale genomic recombination events detected by shallowHRD algorithm[1]. Values greater than 15 are considered positive to homologous recombination deficiency.

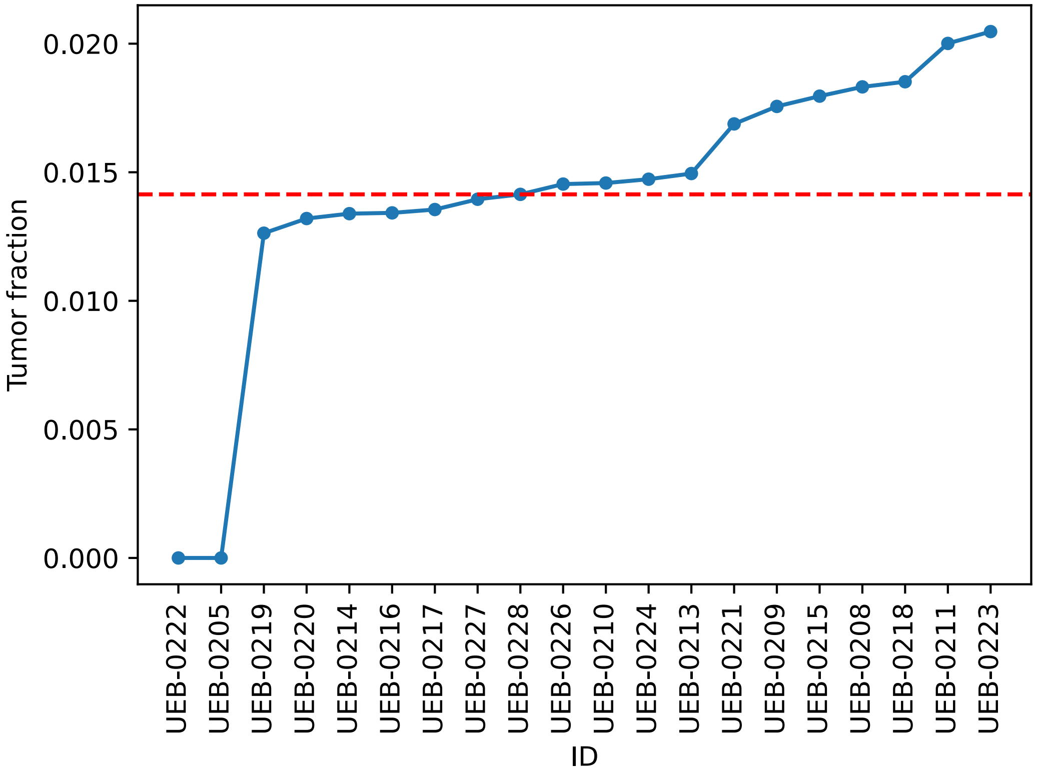

Figure S2. Tumor fraction of cfDNA in shallow WGS in breast cancer patients. The mean tumor fraction detected in this study was 0.014 (range 0-0.02047) evaluated by the ichorCNA algorithm[2].

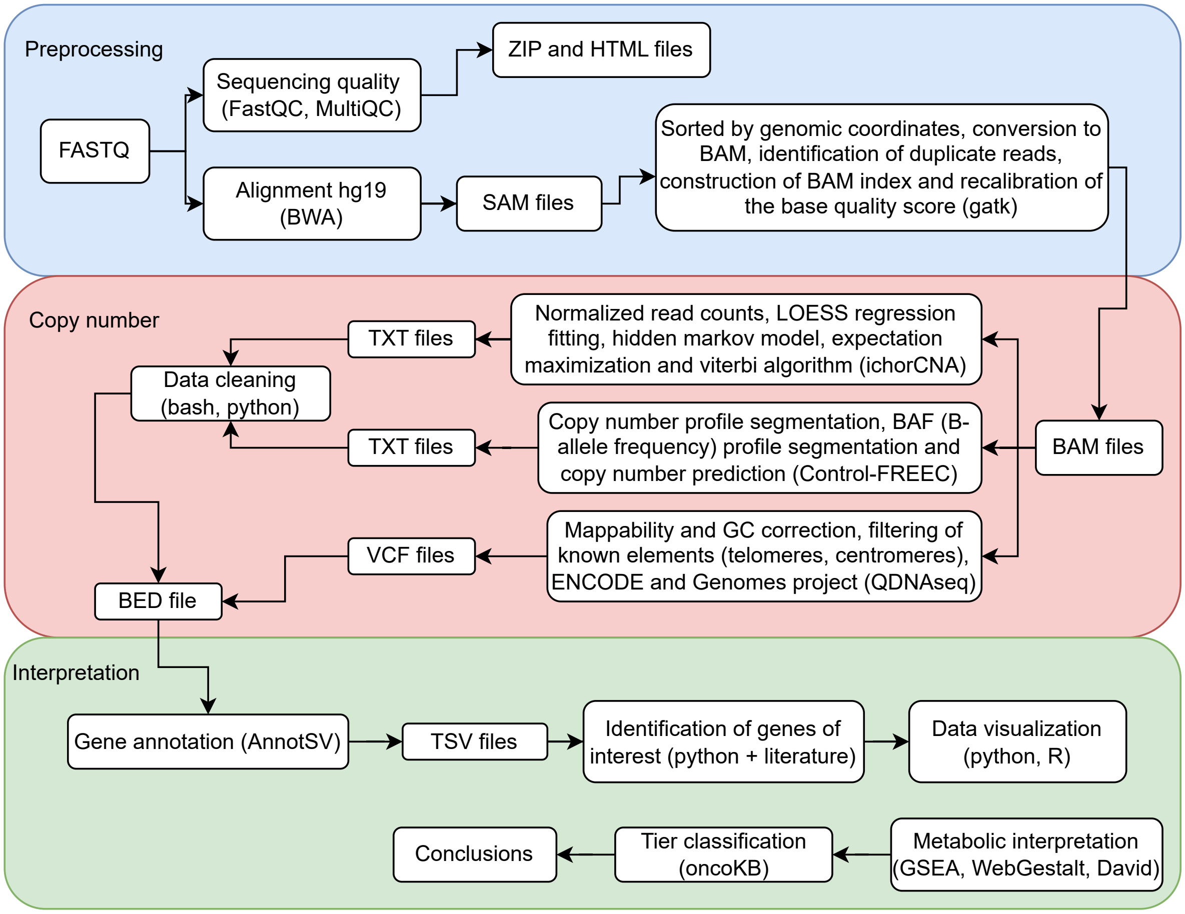

Figure S3. Bioinformatic workflow. The workflow is segmented in three sections. Section one describes the quality evaluation, alignment, and data preprocessing. Section two shows the identification of somatic copy number alterations using three different algorithms. Section three presents interpretation and data visualization.

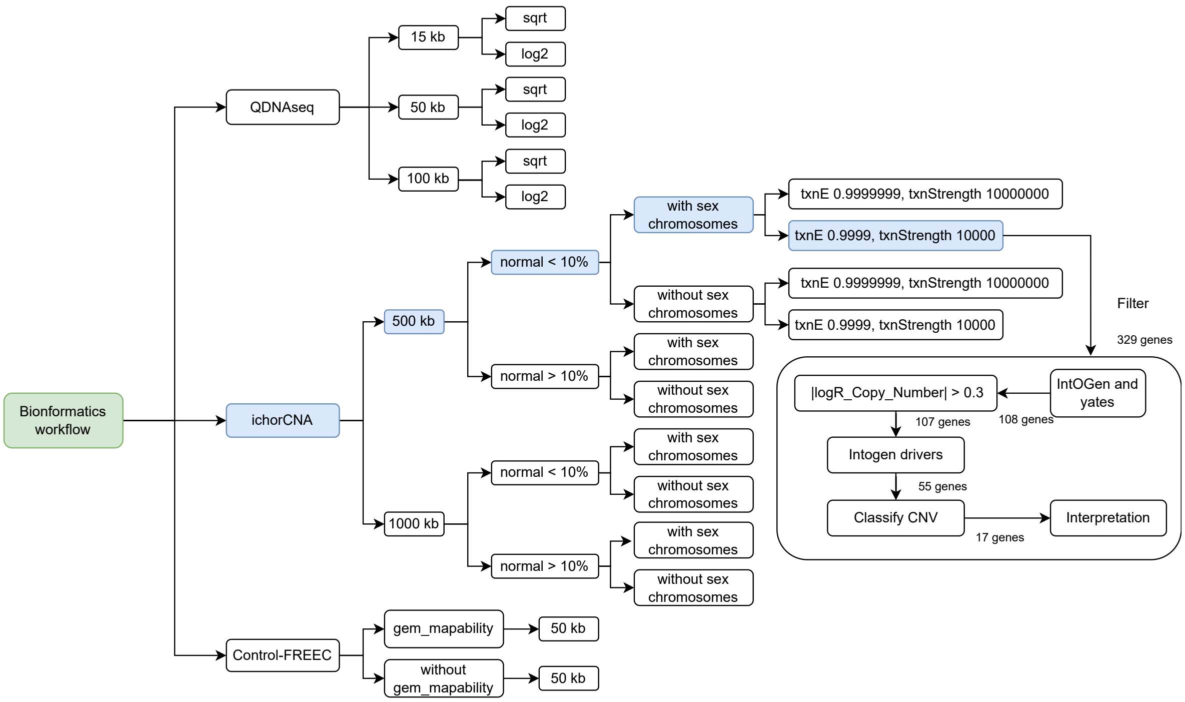

Figure S4. Parameters used in the implementation of algorithms for the somatic copy number alterations.

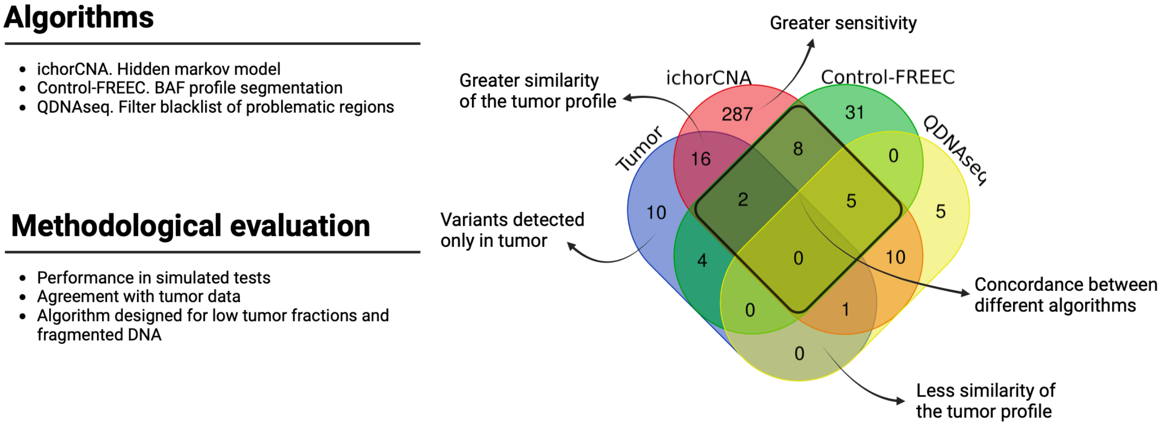

Figure S5. Selection criteria of algorithms to detect somatic copy number alterations. The model and the methodological used for each algorithm (left), and the detection and concordance (right) of SCNA are presented.

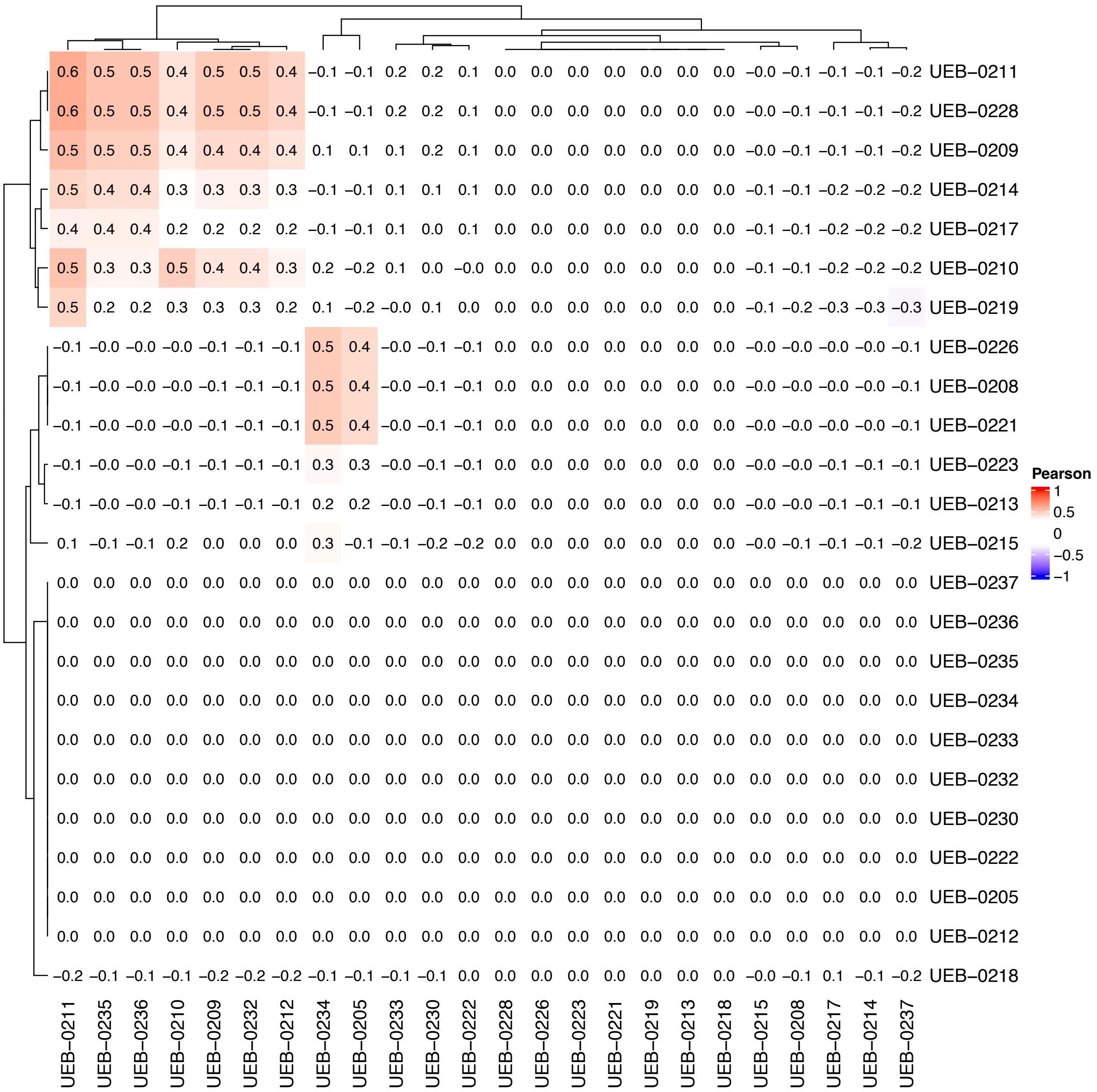

Figure S6. Pearson correlation of somatic copy number alterations between cfDNA and tumor samples.

References

1. Eeckhoutte, A. *et al.* ShallowHRD: detection of homologous recombination deficiency from shallow whole genome sequencing. *Bioinforma. Oxf. Engl.* **36**, 3888–3889 (2020).

2. Adalsteinsson, V. A. *et al.* Scalable whole-exome sequencing of cell-free DNA reveals high concordance with metastatic tumors. *Nat. Commun.* **8**, 1324 (2017).

Table S1

ID	sWGS	Tumor	cfDNA concent	RE	RP	Her2	Molecular Subt	Histopatology	T	N	M	Stage	Sex	Treatment	Age	Family history	Weight (Kg)	Height (m)	BMI (kg/m²)	StatusTreat_cf	StatusTreat_Tu
UEB-0205	Yes	Yes	0.81	1	0.4	Negative	Luminal A	IDC	1	0	0	I	Female	RTx-CTx	76	Yes	55.00	1.68	19	Treated Tumor	Primary tratment
UEB-0208	Yes	Yes	0.88	Positive 80%	Negative	Negative	Luminal B	IDC	1	0	0	I	Female	CTx	55	No	58.00	1.55	24	Primary tratment	Primary tratment
UEB-0209	Yes	Yes	0.36	0.6	0.6	Negative	Luminal A	IDC	2	0	0	II	Female	Sx	51	Yes	61.00	1.40	31	Treated Tumor	Primary tratment
UEB-0210	Yes	Yes	1.27	1	1	Mistaken (++/++	Luminal B Like	IDC	2	0	0	II	Female	RTx-CTx	55	Yes	59.00	1.64	22	Treated Tumor	Treated Tumor
UEB-0211	Yes	Yes	01.07	1	1	Negative	Luminal A	IDC	4	1	0	III	Male	RTx-CTx	68	No	72.00	1.56	30	Treated Tumor	Treated Tumor
UEB-0212	No	Yes	NA	NA	NA	NA	NA	Phyllodes	1	0	0	I	Female	RTx-CTx	62	Unknown	Unknown	Unknown	NA	NA	Primary tratment
UEB-0213	Yes	Yes	0.32	1	1	Negative	Luminal A	Lobular	2	0	0	II	Female	RTx-CTx	42	No	81.00	1.65	30	Primary tratment	Treated Tumor
UEB-0214	Yes	Yes	1.55	NA	NA	NA	NA	Phyllodes	NA	NA	NA	NA	Female	RTx	68	Yes	56.00	1.55	23	Treated Tumor	Primary tratment
UEB-0215	Yes	Yes	0.56	1	0.9	Negative	Luminal A	IDC	4	3	0	III	Female	Sx	61	Yes	70.00	1.65	26	Primary tratment	Primary tratment
UEB-0216	Yes	Yes	0.5	1	1	Negative	Luminal A	IDC	1	0	0	I	Female	RTx-CTx	40	Yes	55.00	1.59	22	Primary tratment	Primary tratment
UEB-0217	Yes	Yes	5.3	Negative	NA	NA	NA	Phyllodes	NA	NA	NA	NA	Female	RTx-CTx	49	Yes	48.00	1.69	17	Primary tratment	Primary tratment
UEB-0218	Yes	Yes	3.18	Negative	NA	Negative	Triple Negative	Metaplastic	4	0	0	III	Female	CTx	83	No	58.00	1.50	26	Primary tratment	Primary tratment
UEB-0219	Yes	Yes	0.49	0.95	96.80%	Negative	Luminal A	IDC	4	0	0	III	Female	RTx	70	Yes	49.00	1.45	23	Primary tratment	Primary tratment
UEB-0220	Yes	Yes	1.32	Negative	NA	Negative	Triple Negative	Metaplastic	3	0	0	II	Female	CTx	59	No	65.00	1.65	24	Treated Tumor	Treated Tumor
UEB-0221	Yes	Yes	1.27	1	0.05	Positive 3+	Luminal B Like	IDC	3	2	0	III	Female	CTx	70	Yes	68.00	1.60	27	Primary tratment	Primary tratment
UEB-0222	Yes	Yes	0.43	1	0.9	Negative	Luminal A	IDC	2	1	0	II	Female	Sx	73	Yes	66.00	1.57	27	Primary tratment	Primary tratment
UEB-0223	Yes	Yes	0.71	1	1	Negative	Luminal A	Mucinous	2	0	0	II	Female	RTx	49	Yes	106.00	1.68	38	Primary tratment	Primary tratment
UEB-0224	Yes	Yes	01.02	1	1	Negative	Luminal A	IDC	2	1	0	II	Female	RTx-CTx	47	Yes	74.00	1.60	29	Primary tratment	Primary tratment
UEB-0225	No	Yes	NA	0.1	0.05	Mistaken (2++)	Luminal B Like	IDC	1	1	0	II	Female	RTx-CTx	56	No	66.00	1.51	29	NA	Treated Tumor
UEB-0226	Yes	Yes	0.64	NA	NA	NA	NA	Phyllodes	NA	NA	NA	NA	Female	Sx	51	Unknown	Unknown	Unknown	NA	Primary tratment	Primary tratment
UEB-0227	Yes	Yes	0.28	1	1	Negative	Luminal A	Lobular	4	0	0	III	Female	RTx	81	No	64.00	1.57	26	Primary tratment	Treated Tumor
UEB-0228	Yes	Yes	0.48	0.95	0.8	Negative	Luminal A	IDC	2	1	0	NA	Female	Sx	60	Unknown	Unknown	Unknown	NA	Unknown	Unknown
UEB-0229	No	Yes	NA	1	0.95	Negative	Luminal A	IDC	2	1	0	II	Female	RTx-CTx	47	Unknown	Unknown	Unknown	NA	NA	Treated Tumor
UEB-0230	No	Yes	NA	0.95	0.4	Mistaken (2+)	Luminal B Like	IDC	0	3	0	III	Female	RTx-CTx	55	Yes	65.00	1.58	26	NA	Treated Tumor
UEB-0231	No	Yes	NA	NA	NA	Mistaken (2+)	HER2 Positive	IDC	4	2	0	III	Female	RTx-CTx	46	Unknown	Unknown	Unknown	NA	NA	Treated Tumor
UEB-0232	No	Yes	NA	0.6	0.9	Mistaken (++/++	Luminal B Like	IDC	4	2	0	III	Male	Sx	72	No	63.00	1.73	21	NA	Primary tratment
UEB-0233	No	Yes	NA	0.8	Negative	Negative	Luminal B	IDC	4	2	0	III	Female	RTx-CTx	40	No	64.00	1.59	25	NA	Treated Tumor
UEB-0234	No	Yes	NA	Negative	Negative	Negative	Triple Negative	IDC	2	2	0	III	Female	RTx-CTx	54	Unknown	Unknown	Unknown	NA	NA	Treated Tumor
UEB-0235	No	Yes	NA	Negative	Negative	Positive (3+)	Luminal B Like	IDC	3	0	0	II	Female	Sx	30	Unknown	Unknown	Unknown	NA	NA	Primary tratment
UEB-0236	No	Yes	NA	Positive	Positive	Negative	Luminal A	IDC	2	1	0	II	Female	RTx-CTx	40	Unknown	Unknown	Unknown	NA	NA	Primary tratment
UEB-0237	No	Yes	NA	0.98	70	Negative (+/+++	Luminal A	IDC	2	1	0	II	Male	RTx-CTx	77	Unknown	Unknown	Unknown	NA	NA	Primary tratment
UEB-0238	No	Yes	NA	Negative	Negative	Weakly Positive	HER2 Positive	IDC	2	0	0	II	Female	RTx-CTx	68	Unknown	Unknown	Unknown	NA	NA	Primary tratment
UEB-0239	No	Yes	NA	0.25	0.01	Positive (3+)	Luminal B Like	IDC	2	0	0	II	Female	Sx	72	Unknown	Unknown	Unknown	NA	NA	Primary tratment
UEB-0240	No	Yes	NA	Negative	Negative	Negative	Triple Negative	IDC	4	1	0	III	Female	RTx-CTx	71	Unknown	Unknown	Unknown	NA	NA	Primary tratment
UEB-0241	No	Yes	NA	NA y 100%	15% y negative	Mistaken (2+) y	Luminal B Like	IDC	2	0	0	II	Female	RTx-CTx	48	Unknown	Unknown	Unknown	NA	NA	Treated Tumor
UEB-0242	No	Yes	NA	0.9	Negative	Positive (3+)	Luminal B Like	Mucinous	4B	1	0	III	Female	RTx-CTx	49	Unknown	Unknown	Unknown	NA	NA	Treated Tumor
UEB-0243	No	Yes	NA	Positive	Positive	Negative	Luminal A	IDC	2	1	0	II	Female	RTx-CTx	53	Yes	70.00	1.52	30	NA	Treated Tumor
UEB-0244	No	Yes	NA	Positive 20%	Positive 20%	Mistaken (2++)	Luminal B Like	IDC	4	1	0	III	Female	RTx-CTx	44	Unknown	Unknown	Unknown	NA	NA	Treated Tumor

Table S2

Cellular process	Transcription factor/regulator	Histone modifier	Genome integrity	RTK signaling	Cell cycle	MAPK signaling	PI(3)K signaling	TGF-β signaling	Wnt/β-catenin signaling	Histone	Proteolysis	Splicing	HIPPO signaling	DNA methylation	Metabolism	NFE2L	Protein phosphatase	Ribosome	TOR signaling	Other
**Gene**	*VHL*<br>*GATA3*<br>*TSHZ3*<br>*EP300*<br>*CTCF*<br>*TAF1*<br>*TSHZ2*<br>*RUNX1*<br>*MECOM*<br>*TBX3*<br>*SIN3A*<br>*WT1*<br>*EIF4A2*<br>*FOXA1*<br>*PHF6*<br>*CBFB*<br>*SOX9*<br>*ELF3*<br>*VEZF1*<br>*CEBPA*<br>*FOXA2*	*MLL3*<br>*MLL3*<br>*ARID1A*<br>*PBRM1*<br>*SETD2*<br>*NSSD1*<br>*SETBP1*<br>*KDM5C*<br>*KDM6A*<br>*MLL4*<br>*ARID5B*<br>*ASXL1*<br>*EZH2*	*TP53*<br>*ATM*<br>*ATRX*<br>*BRCA2*<br>*ATR*<br>*STAG2*<br>*BAP1*<br>*BRCA1*<br>*SMC1A*<br>*SMC3*<br>*CHEK2*<br>*RAD21*<br>*ERCC2*	*EGFR*<br>*FLT3*<br>*EPHA3*<br>*ERBB4*<br>*PDGFRA*<br>*EPHB6*<br>*FGFR2*<br>*KIT*<br>*FGFR3*	*CDKNA2*<br>*RB1*<br>*CDK12*<br>*CDKN1B*<br>*CCND1*<br>*CDKN1A*<br>*CDKN2C*	*KRAS*<br>*NF1*<br>*MAP3K1*<br>*BRAF*<br>*NRAS*<br>*MAP2K4*<br>*MAPK8IP1*	*PIK3CA*<br>*PTEN*<br>*PIK3R1*<br>*TLR4*<br>*PIK3CG*<br>*AKT1*	*SMAD4*<br>*TGFBR2*<br>*ACVR1B*<br>*SMAD2*<br>*ACVR2A*	*APC*<br>*CTNNB1*<br>*AXIN2*<br>*TBL1XR1*<br>*SOX17*	*HIST1H1C*<br>*H3F3C*<br>*HIST1H2BD*	*FBXW7*<br>*KEAP1*<br>*SPOP*	*SF3B1*<br>*U2AF1*<br>*PCBP1*	*CDH1*<br>*AJUBA*	*DNMT3A*<br>*TET2*	*IDH1*<br>*IDH2*	*NEF2L2*<br>*NEF2L3*	*PPP2R1A*<br>*PTPN11*	*RPL22*<br>*RPL5*	*MTOR*<br>*STK11*	*NAV3*<br>*NOTCH1*<br>*LRRK2*<br>*MALAT1*<br>*ARHGAP35*<br>*POLQ*<br>*NCOR1*<br>*USP9X*<br>*NPM1*<br>*HGF*<br>*EPPK1*<br>*AR*<br>*LIFR*<br>*PRX*<br>*CRPAK*<br>*EGR3*<br>*B4GAL53*<br>*MIR142*
